# Fermented *Protaetia brevitarsis* Larvae Ameliorates Chronic Ethanol-Induced Hepatotoxicity in Mice via AMPK and TLR-4/TGF-β1 Pathways

**DOI:** 10.4014/jmb.2310.10003

**Published:** 2023-11-14

**Authors:** Hyo Lim Lee, Jong Min Kim, Min Ji Go, Seung Gyum Joo, Tae Yoon Kim, Han Su Lee, Ju Hui Kim, Jin-Sung Son, Ho Jin Heo

**Affiliations:** 1 Division of Applied Life Science (BK21), Institute of Agriculture and Life Science, Gyeongsang National University, Jinju 52828, Republic of Korea; 2 HMO Health Dream Agricultural Association Corporation, Republic of Korea

**Keywords:** *Protaetia brevitarsis*, inflammation, alcoholic liver disease

## Abstract

This study evaluated the hepatoprotective effect of fermented *Protaetia brevitarsis* larvae (FPB) in ethanol-induced liver injury mice. As a result of amino acids in FPB, 18 types of amino acids including essential amino acids were identified. In the results of in vitro tests, FPB increased alcohol dehydrogenase (ADH) and aldehyde dehydrogenase (ALDH) activities. In addition, FPB treatment increased cell viability on ethanol- and H_2_O_2_-induced HepG2 cells. FPB ameliorated serum biomarkers related to hepatoxicity including glutamic oxaloacetic transaminase, glutamine pyruvic transaminase, total bilirubin, and lactate dehydrogenase and lipid metabolism including triglyceride, total cholesterol, high-density lipoprotein cholesterol, and low-density lipoprotein cholesterol. Also, FPB controlled ethanol metabolism enzymes by regulating the protein expression levels of ADH, ALDH, and cytochrome P450 2E1 in liver tissue. FPB protected hepatic oxidative stress by improving malondialdehyde content, reduced glutathione, and superoxide dismutase levels. In addition, FPB reversed mitochondrial dysfunction by regulating reactive oxygen species production, mitochondrial membrane potential, and ATP levels. FPB protected ethanol-induced apoptosis, fatty liver, and hepatic inflammation through p-AMP-activated protein kinase and TLR-4/NF-κB signaling pathways. Furthermore, FPB prevented hepatic fibrosis by decreasing TGF-β1/Smad pathway. In summary, these results suggest that FPB might be a potential prophylactic agent for the treatment of alcoholic liver disease via preventing liver injury such as fatty liver, hepatic inflammation due to chronic ethanol-induced oxidative stress.

## Introduction

Various alcoholic beverages that have been consumed for thousands of years are a major cause of the morbidity and mortality of alcoholic liver disease (ALD) [1]. Alcohol absorbed into the body is diffused into several tissues in the body, and alcohol concentration increases rapidly in vascular-rich tissues such as the brain, lung, and liver [2]. In particular, the liver is the main organ of alcohol metabolism, and more than 90% of alcohol in the blood undergoes oxidative metabolism in the liver [3]. Three enzyme systems are mainly involved in the alcohol metabolism process. Most of the alcohol is metabolized by alcohol dehydrogenase (ADH) pathway and decomposed by the microsomal ethanol oxidizing system (MEOS) when consumed in excess or chronically [2]. Finally, the catalase pathway mediates alcohol oxidation, and its contribution to alcohol metabolism and clinical significance is little [4]. Excessive reactive oxygen species (ROS) produced by ethanol consumption cause hepatotoxicity and reduce endogenous antioxidants such as superoxide dismutase (SOD) and glutathione (GSH) in the liver [5, 6]. When the antioxidant system that removes ROS is destroyed, the B-cell lymphoma (Bcl)-2 family mediated apoptosis pathway is activated due to mitochondrial adenosine triphosphate (ATP) production, mitochondrial membrane potential reduction, and ROS accumulation due to ethanol-induced oxidative stress [7]. Furthermore, the liver damaged by ethanol toxicity can influence hepatic lipid metabolism, and acetaldehyde is decomposed by aldehyde dehydrogenase (ALDH) to water and carbon dioxide or converted to fatty acids and accumulated in the liver in the form of triglycerides, causing fatty liver [8]. Also, chronic ethanol consumption can decrease the expression of p-AMP-activated protein kinase (AMPK), which plays an important role in energy homeostasis, thereby inhibiting lipid oxidation and increasing cholesterol and fatty acid synthesis [9]. In addition, when the permeability of colon blood vessels increases due to chronic ethanol consumption, the number of microbial-derived endotoxins increases and moves to the liver through the bloodstream [10]. Lipopolysaccharide (LPS) as endotoxin introduced into the liver that bind to Toll-like receptor (TLR)-4 on the surface of Kupffer cells (KCs) and initiates the secretion of inflammatory cytokines such as tumor necrosis factor (TNF)-α, interleukin (IL)-6, IL-1β [10]. Therefore, a large amount of ROS and toxic substances produced during alcohol metabolism can destroy the antioxidant system and mitochondrial function and cell survival system, and eventually reach fatty liver and hepatitis [3]. Ultimately, the activation of hepatic stellate cells due to the accumulation of ROS and the increase of inflammatory cytokines leads to abnormal proliferation of the extracellular matrix (ECM), which can lead to hepatic fibrosis [1].

Recently, edible insects have been attracting attention as future alternative food because the average life expectancy of humans is improving due to changes in diet and the development of medical technology around the world [11]. The UN Food and Agriculture Organization (FAO) predicts that the world's population will grow to 9.7 billion by 2050, increasing the demand for food due to population growth. Accordingly, the FAO is focusing on edible insects as a new food substitute for the future [12]. Edible insects have been used in herbal medicine since ancient times, and because they have a high protein content, they are nutritionally excellent and have the advantage of being able to be mass produced in a short generation cycle [13]. The *Protaetia brevitarsis* larvae are classified as the beetle family and are mainly distributed in Korea, Japan, Taiwan, China, and Europe [14]. As a functional material, *P. brevitarsis* larvae have been reported to have various physiological activities such as anti-obesity, antioxidant, anti-inflammation, and anti-neoplastic effects in various in vitro and in vivo studies [14-17]. However, research on the mechanism of its protective effects on ethanol-induced liver toxicity is still insufficient. In addition, there are studies that have experimented the activity of *P. brevitarsis* larvae with various kinds of pretreatment analysis such as solvent extraction, protein hydrolysis, and fer-mentation of useful microbes [15-18]. However, there are few studies using *P. brevitarsis* larvae fermented with *Bacillus subtilis* after protein hydrolysis. A previous study reported that lyophilized *P. brevitarsis* larvae protected against liver damage in diethyl nitrosamine-induced hepatotoxic mice [17]. Since low-molecular proteins are advantageous for nutritional and bioactivity effects along with having high absorption rates, it is important to decompose the amino acids from high-molecular to low-molecular through fermentation [19,20]. In order to increase the amino acid absorption rate by low molecularization of the protein, *P. brevitarsis* larvae were hydrolyzed and then fermentation with *B. subtilis*. Therefore, in this study, the mechanism of the hepaprotective effect of fermented *P. brevitarsis* larvae was confirmed in chronic ethanol consumption mice.

## Materials and Methods

### Reagents

β-Nicotinamide adenine dinucleotide (NAD^+^), ADH, ALDH, Minimum Essential Medium Eagle (MEM), fetal bovine serum (FBS), penicillin, streptomycin, dimethyl sulfoxide (DMSO), 3-(4,5-dimethylthiazol-2-yl)-2,5-diphenyltetrazolium bromide (MTT), 2,7-dichlorofluoroscin diacetate (DCF-DA), 2-thiobarbituric acid (TBA), trichloroacetic acid (TCA), phenylmethanesulfonyl fluoride (PMSF), pyruvic acid, malic acid, mannitol, egtazic acid (EGTA), hydroxyethyl piperazine ethane sulfonic acid (HEPES), and 5,5',6,6'-tetrachloro-1,1',3,3'-tetraethylbenzimidazolocarbocyanine iodide (JC-1) were purchased from Sigma-Aldrich Chemical Co. (USA). Ethanol was purchased from Ethanol Supplies World Co., Ltd. (Republic of Korea). Ethanol and acetaldehyde assay kits were purchased from Megazyme (Ireland).

### Sample Preparation

The fermented *P. brevitarsis* larvae powder product used in the experiment was supplied by HMO Health Dream (Taean, Republic of Korea). One hundred grams of *P. brevitarsis* larvae were dissolved in 1 L of distilled water and hydrolyzed by proteases including papain and bromelain (Bision Corp., Republic of Korea). Then, it was incubated with 2% (v/v). *B. subtilis* KCTC 1428BP (1 × 10^5^ CFU/ml) at 30°C for 48 h. The fermented *P. brevitarsis* larvae (FPB) were evaporated under reduced pressure and lyophilized. Prepared samples were stored at -20°C until further use.

### Analysis of Amino Acid

Powdered FPB mixed with 6 N HCl was vacuum-sealed and hydrolyzed in a heating block (110 ± 1°C) for 24 h. Then, HCl was removed from the extract using a rotary vacuum evaporator at 40°C, washed three times with distilled water, and concentrated under reduced pressure condition. The hydrolysate was dissolved in 0.2 N sodium citrate buffer (pH 2.2) and filtered with 0.22 μm membrane filter (Jiangsu Green Union Science Instrument Co., Ltd., China). The mixture was analyzed using auto amino acid analyzer (L-8900, Hitachi High Technologies Corp., Japan). The experimental information of amino acid analysis is presented as follows: The column used a Hitachi HPLC packed column with ionexchange resin, and the flow rate and buffer solution are set to 25 ml/h of ninhydrin and pH 3.2-10.0 respectively. The column temperature and reaction temperature were set at 46°C and 88°C, respectively. The essential amino acids in the FPB were calculated by amino acid score (AAS), which was detected by comparing the ratio of contents of each essential amino acid to the joint FAO/World Health Organization (WHO)/United Nations University (UNU) amino acid recommendations [21].

### In Vitro ADH and ALDH Activities

To confirm the ADH and ALDH activities of FPB, it was determined using a method described by Bostian and Betts [22] with some modifications. To measure the ADH activity, 20 mM NAD^+^, 1 M-Tris buffer (pH 8.8), 0.2 M ethanol, and FPB samples were mixed and reacted at room temperature for 10 min. Then, 5 U/ml of ADH was added, reacted at room temperature for 10 min, and the absorbance was determined at 340 nm using a microplate reader (Epoch 2, BioTek Instruments, Inc., USA).

To measure the ADH activity, 20 mM NAD^+^, 1 M-Tris buffer (pH 8.0), 1 M acetaldehyde, 3 M KCl, 0.33 M 2-mercaptoethanol, and FPB samples were mixed and reacted at room temperature for 10 min. Then, 1 U/ml of ALDH was added, reacted at room temperature for 10 min, and the absorbance was determined at 340 nm using a microplate reader (Epoch 2).

### Cell Culture

HepG2 cells were obtained from the Korean Collection for Type Cultures (KCTC, Republic of Korea) and cultured in MEM media containing 10% FBS, 50 unit/ml of penicillin, and 100 μg/ml of streptomycin. The cells were incubated in an incubator maintained at 5% CO_2_ and 37°C.

### Cell Viability

To determine the cell viability, it was assessed using the MTT method [23]. HepG2 cells were seeded on 96-well plate at a density of 1 × 10^4^ cells/well for 24 h. Cells were treated with different concentrations of FPB for 30 min, and then 500 mM ethanol was added. After 24 h incubation, the 5 mg/ml of MTT stock solution was reacted for 3 h. Media were removed, and the produced MTT formazan crystals were dissolved in DMSO. The formed formazan was measured at wavelengths of 570 and 620 nm using a microplate reader (Epoch 2).

### Animal Experimentation

**Acute ethanol-induced mice model.** C57BL/6 mice (4 weeks old, male, *n* = 5) were purchased from Samtako (Republic of Korea), and the mice were housed under standard laboratory conditions of 12/12 h light/dark cycles at 22 ± 2°C. After adaptation for a week, the mice were randomly divided into mice. To confirm the ethanol metabolic effect of FPB, the mice were assigned to 4 groups: control group, ethanol group, FPB50 (50 mg/kg of body weight/day), and FPB100 (100 mg/kg of body weight/day) groups. FPB and ethanol groups were treated with FPB or drinking water by oral gavage, respectively. After 30 min, 5 g/kg of body weight/day of 25% (v/v) ethanol was orally administered except control group. The dose of concentration of ethanol and FPB were determined from previous studies [15, 23]. Blood was taken from the abdominal aorta 0, 30, 60, 90, 120, and 180 min after oral administration of ethanol. After, the collected blood samples were centrifuged at 13,000 ×*g* for 10 min and ethanol and acetaldehyde concentration were immediately detected in serum using commercial kits (Megazyme, Ireland).

**Chronic ethanol-induced mice model.** Additionally, in order to confirm the improvement effect of FPB on chronic ethanol-induced mice, C57BL/6 mice also were used (*n* = 15). The chronic ethanol treatment mice were randomly divided into 4 groups, and treated each diet by oral gavage for 8 weeks as follows. The control group mice were treated with drinking water, ethanol group mice were treated with ethanol (25% v/v, 5 g/kg of body weight/day). The FPB 50 and FPB 100 groups mice were fed FPB 50 mg/kg of body weight/day and FPB 100 mg/kg of body weight/day, respectively prior to ethanol treatment. Mice were anesthetized by CO_2_ and obtained blood samples obtained from the abdominal aorta were rapidly centrifuged at 13,000 ×*g* for 10 min to obtain the serum. All animal experimental procedures were approved by the Institutional Animal Care and Use Committee (IACUC) of Gyeongsang National University guidelines (Certificate No. GNU-220710-M0079) on 10 July 2022.

### Serum Biochemicals Analysis

The glutamic oxaloacetic transaminase (GOT), glutamine pyruvic transaminase (GPT), γ-glutamyl transferase (GGT), total bilirubin (TBIL), lactate dehydrogenase (LDH), triglyceride (TG), total cholesterol (TCHO), and high-density lipoprotein cholesterol (HDLC) were measured using clinical chemistry analyzer (Fuji dri-chem 4000i; Fuji film Co., Japan). Low-density lipoprotein cholesterol (LDLC) content and ratio of HDLC to TCHO (HTR) were calculated as follows: LDLC (mg/dL) = TCHO – (HDLC + TG/5), HTR (%) = (HDLC/TCHO) ×100.

### Antioxidant System Activity

**Malondialdehyde (MDA) content.** To confirm the MDA content in liver tissue, it was analyzed using a method described by Ghani *et al*. [24]. The homogenized liver tissue with phosphate-buffered saline (PBS) was centrifuged at 2,450 ×*g* for 10 min. The supernatant was reacted with 1% (v/v) phosphoric acid and 0.67% (v/v) TBA at 95°C for 1 h. The reactant was centrifuged at 600 ×*g* for 10 min to obtain a supernatant, and the absorbance of supernatants was detected at 532 nm using spectrophotometer (UV-1800, Shimadzu, Japan).

**Reduced GSH level.** To determine the reduced GSH Level in liver tissue, it was detected using a method described by Hissin and Hilf [25]. The homogenized liver tissue was mixed with 10 mM phosphate buffer (pH6.0) including 1 mM ethylenediaminetetraacetic acid (EDTA) was centrifuged at 10,000 ×*g* for 15 min at 4°C. This supernatant was mixed with 5% metaphosphoric acid and centrifuged at 2,000 ×*g* for 2 min. The supernatant was reacted with 0.26 M Tris-HCl (pH 7.8), 0.65 N NaOH, and 1 mg/mL of *o*-phthaldialdehyde at room temperature in dark conditions for 15 min. The reactant fluorescence was measured using a fluorescence microplate reader (Infinite 200) at 320 nm (excitation wave) and 420 nm (emission wave).

**SOD level.** To measure the SOD level, the homogenized liver tissue with PBS was centrifuged at 400 ×*g* for 10 min. Cell extraction buffer (10% SOD buffer, 0.4% Triton X-100, and 200 μM PMSF) was added in the obtained pellet. The extract was placed on ice for 30 min and centrifuged at 10,000 ×*g* for 10 min. Then, the obtained supernatant was measured using a SOD kit (Dojindo Molecular Technologies, USA) following the manufacturer’s instructions.

### Mitochondrial Activity

**ROS level.** Isolation of mitochondria from brain tissues was performed as described previously [26]. The ROS production in mitochondria was determined using a respiration buffer (125 mM potassium chloride, 20 mM HEPES, 2.5 mM malate, 2 mM potassium phosphate, 5 mM pyruvate, 1 mM magnesium chloride and 500 μM EGTA, pH 7.0). A mitochondrial extraction was incubated with 25 μM DCF-DA in respiration buffer at room temperature for 20 min. Fluorescence was measured at a wavelength of 530 nm (excitation) and 590 nm (emission)(Infinite 200, Tecan Co., USA).

**Mitochondrial membrane potential.** mitochondrial membrane potential was conducted using the isolated mitochondria with a 200 μM JC-1. The assay buffer contained mitochondrial isolation buffer with 5 mM pyruvate and 5 mM malate. The assay buffer and mitochondrial extraction were added to the wells of a 96-well black plate, followed by the addition of 1 μM JC-1 and mixed gently. The microplate was covered with aluminum foil and left at room temperature for 20 min. Fluorescence (excitation wavelength: 530 nm, emission wavelength: 590 nm) was then measured (Infinite 200, Tecan Co.).

**ATP Content.** The previously preprocessed sample was centrifuged at 13,000 ×*g* for 10 min. The ATP level was measured using a commercial kit (Promega, USA) with a luminescence meter (GloMax-Multi, Promega).

### Western Blot

To confirm the alteration of relative protein expression level caused by ethanol and FPB treatment, western blot was conducted. Liver tissue was homogenized with cold lysis buffer (ProtinEx Animal cell/tissue, GeneAll Biotechnology, Republic of Korea) containing 1% protease inhibitor cocktails (Thermo Fisher Scientific, USA). The homogenates were centrifuged at 13,000 ×*g* at 4°C for 10 min. To load the same concentration of each homogenized tissue, the protein concentration of the supernatant was quantified using Bradford reagent (Bio-Rad, USA) methods with bovine serum albumin as a standard [27]. The samples quantified in equal amounts (25-50 μg) of total protein were separated on SDS polyacrylamide gel and transferred onto a PVDF membrane (Millipore, USA). The membrane was blocked by 5% skim milk and washed in Tris-buffered saline containing 0.1% Tween 20 (TBST). After three times washing, the membrane was incubated in TBST containing primary antibody (dilution of 1:1,000) overnight at 4°C. Then, the membrane was reacted with TBST containing secondary antibody (dilution of 1:3,000) at room temperature for 1 h. The intensity of bands obtained in enhanced chemiluminescence (ECL, Translab, Korea)-exposed membrane was measured with an iBright CL1000 imager (Thermo Fisher Scientific). Band intensity was calculated as the grey value using Image J software (National Institutes of Health, USA) and used β-actin as the loading control. Antibody details are presented in Table 1.

### Statistical Analysis

All data were presented as mean ± standard deviation (SD). Statistical analysis was analyzed using one-way analysis of variance followed by Duncan’s multiple range test by SAS program (Ver. 9.4 SAS Institute, USA). Statistical difference (*p* < 0.05) of each group was shown by different lowercase letters.

## Results

### Amino Acids Composition and Amino Acid Score in FPB

As shown in Table 1, the contents of essential amino acids as histidine, isoleucine, leucine, lysine, threonine, tryptophan, valine, methionine, arginine, and phenylalanine were detected from FPB as 102.02, 14.40, 22.52, 40.73, 14.24, 5.24, 21.14, 5.09, 35.18, and 15.65 mg/g, respectively. Moreover, it was detected that the contents of other non-essential amino acids tyrosine, glycine, serine, alanine, glutamic acid, aspartic acid, proline, and cysteine were 27.12, 24.42, 23.42, 18.64, 52.89, 29.72, 47.42, and 5.88 mg/g, respectively. In addition, the results of AAS to confirm amino acid quality, histidine, isoleucine, leucine, lysine, threonine, tryptophan, valine, methionine + cysteine, and phenylalanine + tyrosine were 165.00, 99.67, 80.00, 188.33, 129.67, 186.33, 112.00, 100.33, and 228.33, respectively.

### In Vitro ADH and ALDH Activities of FPB

**Fig. 1:**
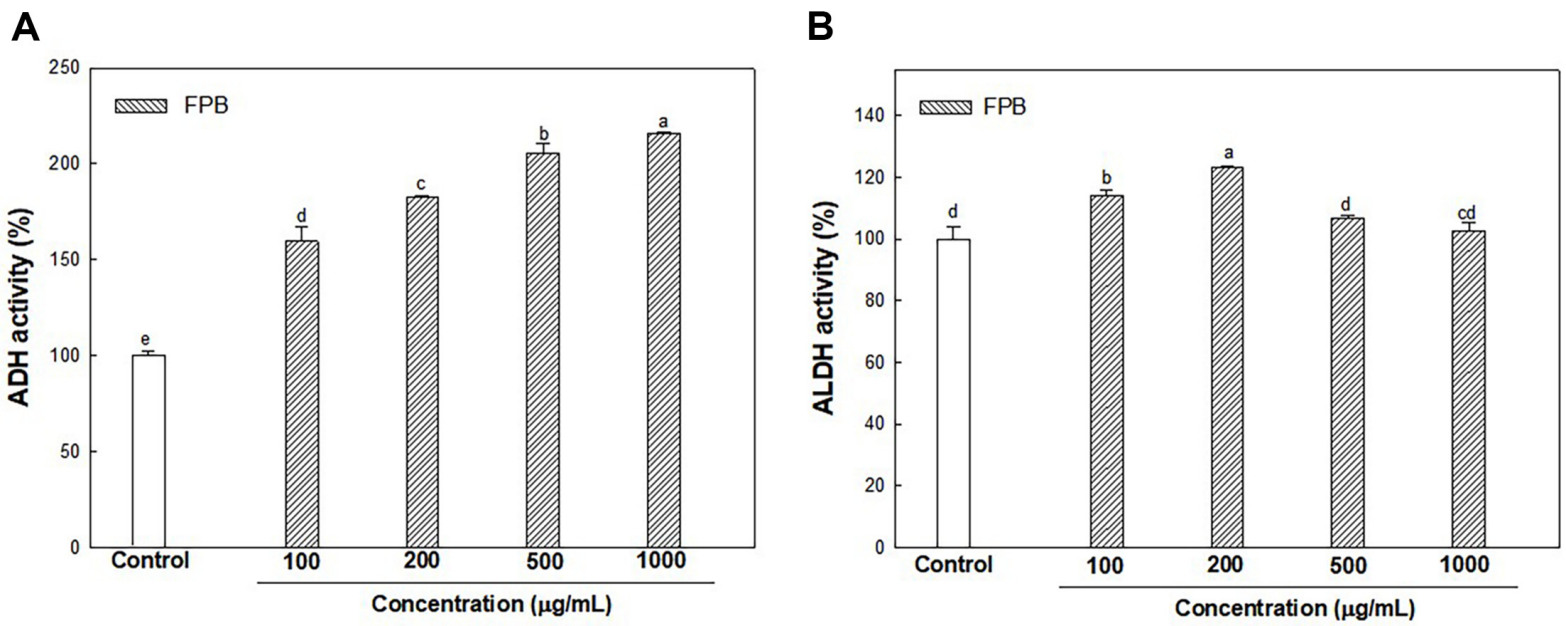
In vitro alcohol dehydrogenase (ADH) (A) and acetaldehyde dehydrogenase (ALDH) (B) activities of fermented *Protaetia brevitarsis* larvae (FPB). Results are presented as mean ± SD (*n* = 3). Data were statistically considered at *p* < 0.05, and different small letters represent the statistical differences.

As shown in Fig. 1, the ADH activity of FPB was detected as 159.65%, 182.51%, 205.86%, and 215.66% at concentrations of 100, 200, 500, and 1,000 μg/mL, respectively, compared with control (100.00%). The ALDH activity of FPB was detected as 114.18%, 123.07%, 106.77%, and 102.65% at concentrations of 100, 200, 500, and 1,000 μg/mL, respectively, compared with control (100.00%).

### Cytoprotective Effect of FPB on HepG2 Cells

**Fig. 2:**
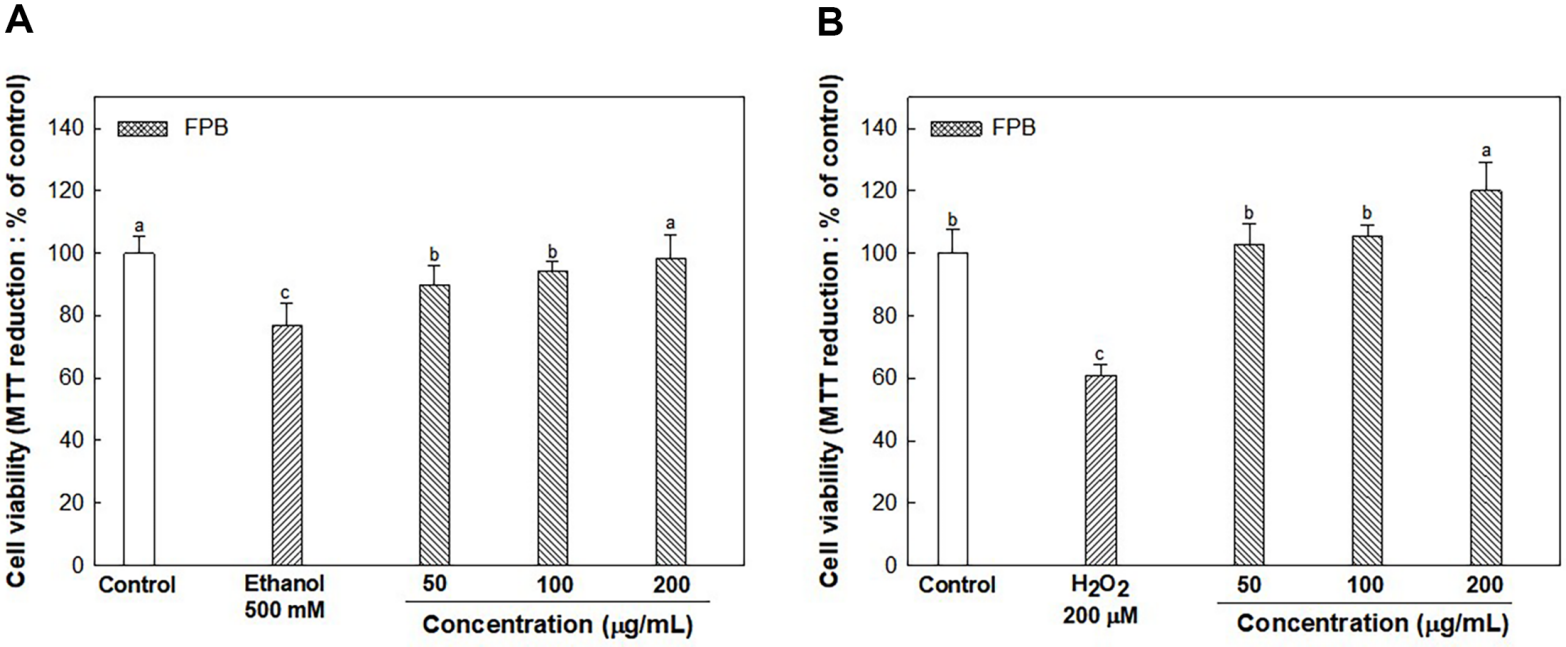
Cytoprotective effect of fermented *Protaetia brevitarsis* larvae (FPB) on HepG2 cells. Cell viability on Ethanol (**A**) and H_2_O_2_- (**B**) induced HepG2 cells. Results are presented as mean ± SD (*n* = 5). Data were statistically considered at *p* < 0.05, and different small letters represent the statistical differences.

As shown in Fig. 2, the cell viability of ethanol (76.91%) and H_2_O_2_ (60.96%) treated group as the negative groups were decreased compared with control group (100.00%). However, FPB-treated groups increased (98.39% and 120.05%, respectively) at concentration of 200 μg/ml compared with negative group.

### Effect of FPB on Serum Ethanol and Acetaldehyde Concentrations in Acute Ethanol Exposure Model

**Fig. 3:**
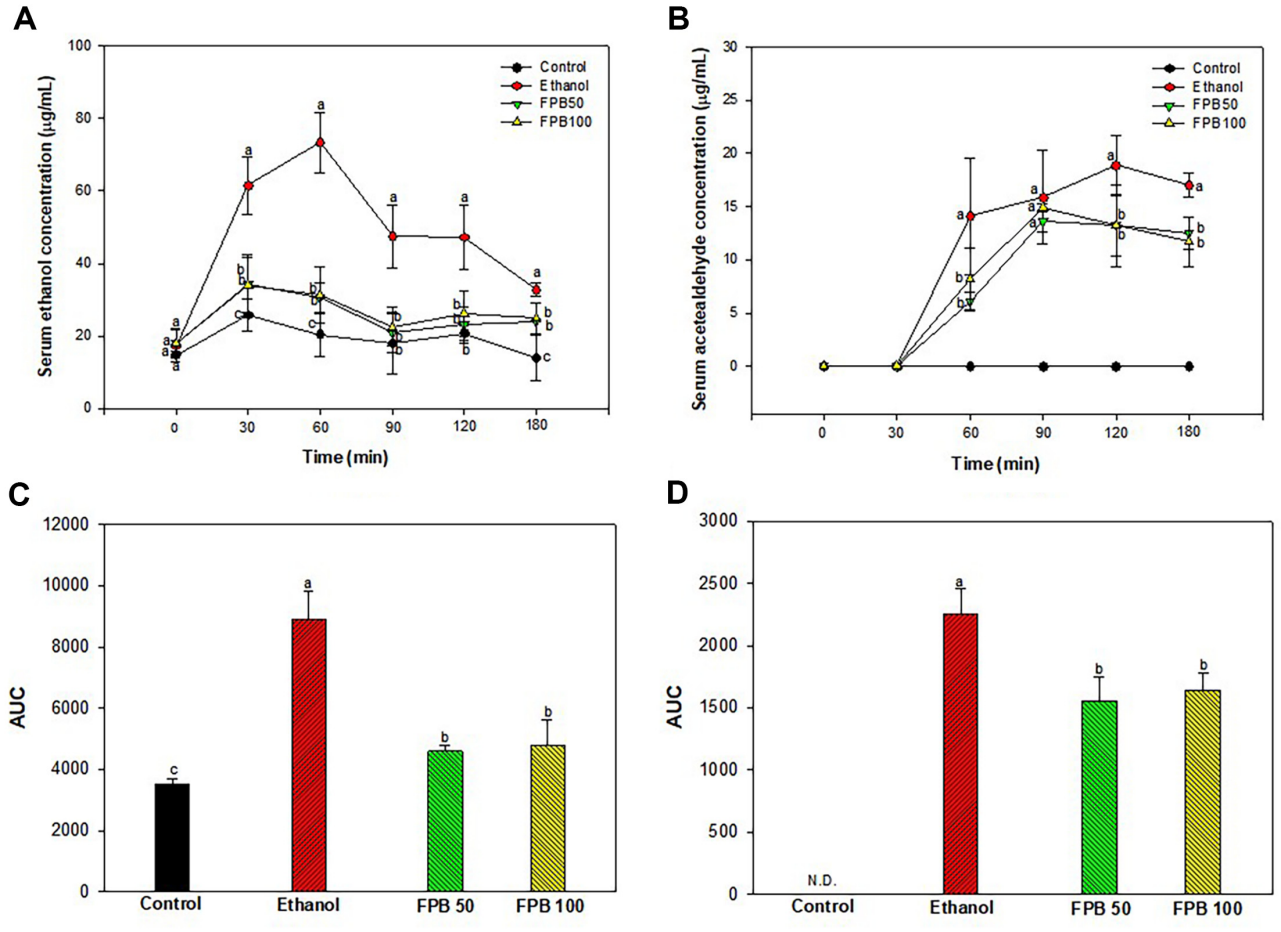
Effect of fermented *Protaetia brevitarsis* larvae (FPB) on acute ethanol-induced mice. Serum ethanol (**A**), acetaldehyde concentration (**B**), area under the curve (AUC) of ethanol concentration (**C**), and AUC of acetaldehyde (**D**) in mice with a single oral dose of ethanol. Results are presented as mean ± SD (*n* = 5). Data were statistically considered at *p* < 0.05, and different small letters represent the statistical differences.

As shown in Fig. 3, the concentrations of ethanol and acetaldehyde in the serum at 0, 30, 60, 90, 120, and 180 min after ethanol administration were presented. At 30 min after administration of ethanol, serum ethanol concentration was elevated rapidly in the ethanol group (61.52 μg/ml) compared with the control group (2.5 μg/ml) (Fig. 3A). Also, the ethanol concentration was the highest in the ethanol group (73.35 μg/ml) at 60 min after administration of ethanol. However, the serum ethanol concentration of the FPB50 and FPB100 groups were decreased (30.54 and 31.31 μg/ml, respectively) compared with the ethanol group at 60 min after administration of ethanol.

Serum acetaldehyde concentration was increased rapidly in the ethanol group at 60 min (14.08 μg/ml) after administration of ethanol and was highest at 120 min (18.88 μg/ml) after administration of ethanol (Fig. 3B). However, the serum acetaldehyde concentration of the FPB50 and FPB100 groups were decreased (13.21 and 13.21 μg/ml, respectively) compared with the ethanol group at 120 min after administration of ethanol. Serum acetaldehyde was not detected in the control group.

Moreover, serum ethanol and acetaldehyde concentrations were detected at 0, 30, 60, 90, 120, and 180 min, and the above experimental results were expressed as area under the curve (AUC) (Fig. 3C and 3D). The AUC of ethanol was reduced in the FPB50 and FPB100 groups (4,590.76 and 4,768.96 μg/ml × min, respectively) compared with the ethanol group (8,916.83 μg/ml × min). The AUC of acetaldehyde was decreased in the FPB50 and FPB100 groups (1,556.56 and 1,638.93 μg/ml × min, respectively) compared with the ethanol group (2,256.50 μg/ml × min).

### Effect of FPB on Serum Biomarkers

As shown in Table 3, the levels of serum biomarkers related to hepatic toxicity are presented. Ethanol treatment (55.60 U/L, 40.60 U/L, 0.60 mg/dL, and 239.67 U/L, respectively) caused an increase in GOT, GPT, TBIL, and LDH levels compared to the control group (43.40 U/L, 31.20 U/L, 0.23 mg/dL, and 127.75 U/L, respectively). However, FPB consumption (FPB50: 42.40 U/L, 27.40 U/L, 0.25 mg/dL, and 165.50 U/L; FPB100: 42.40 U/L, 28.40 U/L, 0.23 mg/dL, and 133.25 U/L, respectively) decreased the level of GOT, GPT, TBIL and LDH compared with the ethanol group. There was no significant difference in serum GGT level between all groups.

As shown in Table 4, the levels of serum biomarkers related to lipid accumulation are presented. Ethanol treatment (161.40, 116.80, 88.40, and 60.68 mg/dL, respectively) induced an increase in TG, TCHO, HDLC, and LDLC levels compared with the control group (147.20, 109.80, 86.40, and 52.84 mg/dL, respectively). However, FPB consumption (FPB50: 113.00, 90.80, 74.40, and 39.00 mg/dL; FPB100: 126.00, 97.80, 82.80, and 40.20 mg/dL, respectively) decreased the level of TG, TCHO, HDLC, and LDLC compared with the ethanol group. HTR (%) was lower in the ethanol group (75.83%) than control group (78.41%). However, FPB50 and FPB100 groups significantly increased HTR (81.76% and 84.87%, respectively).

### Effect of FPB on Antioxidant Parameters

**Fig. 4:**
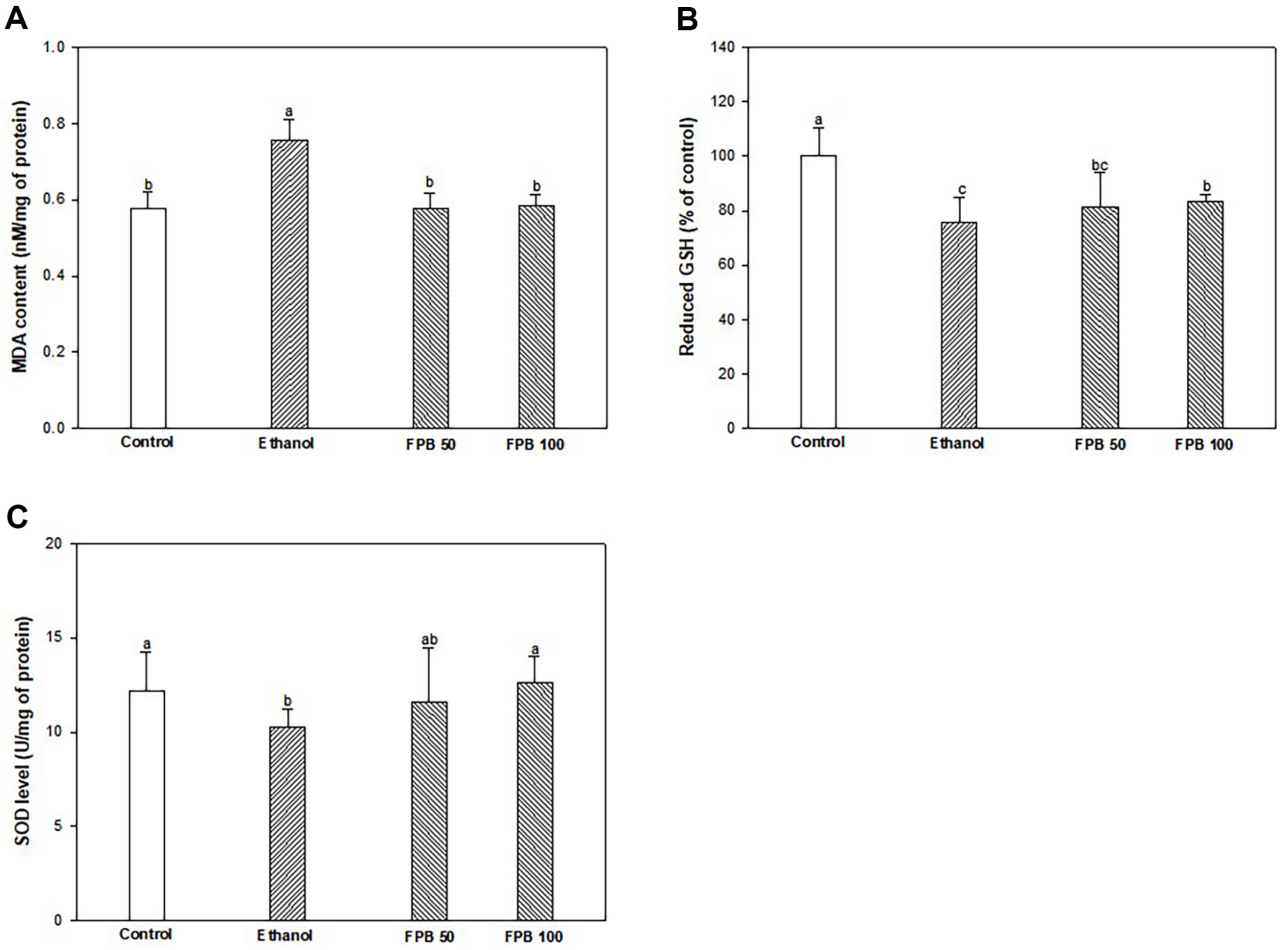
Effect of fermented *Protaetia brevitarsis* larvae (FPB) on antioxidant parameters in ethanol-induced mice. Malondialdehyde (MDA) production (**A**), reduced glutathione (GSH) (**B**), and superoxide dismutase (SOD) level (**C**) in liver tissues. Results are presented as mean ± SD (*n* = 10). Data were statistically considered at *p* < 0.05, and different small letters represent the statistical differences.

As shown in Fig. 4A, the MDA content was increased in the ethanol group (0.76 nM/mg of protein) compared with the control group (0.58 nM/mg of protein). However, the MDA content of the FPB50 and FPB100 groups (0.58 and 0.58 nM/mg of protein, respectively) were reduced compared with ethanol group. Reduced GSH level was reduced in the ethanol group (75.47%) compared to control group (100.00%). However, the reduced GSH level of the FPB 100 group (83.13%) was increased compared with the ethanol group (Fig. 4B). SOD level was reduced in the ethanol group (10.26 U/mg of protein) compared to the control group (12.17 U/mg of protein). However, the SOD levels of the FPB50 and FPB100 groups (11.61 and 12.59 U/mg protein, respectively) were increased compared with the ethanol group (Fig. 4C).

### Effect of FPB on Mitochondrial Functions

**Fig. 5:**
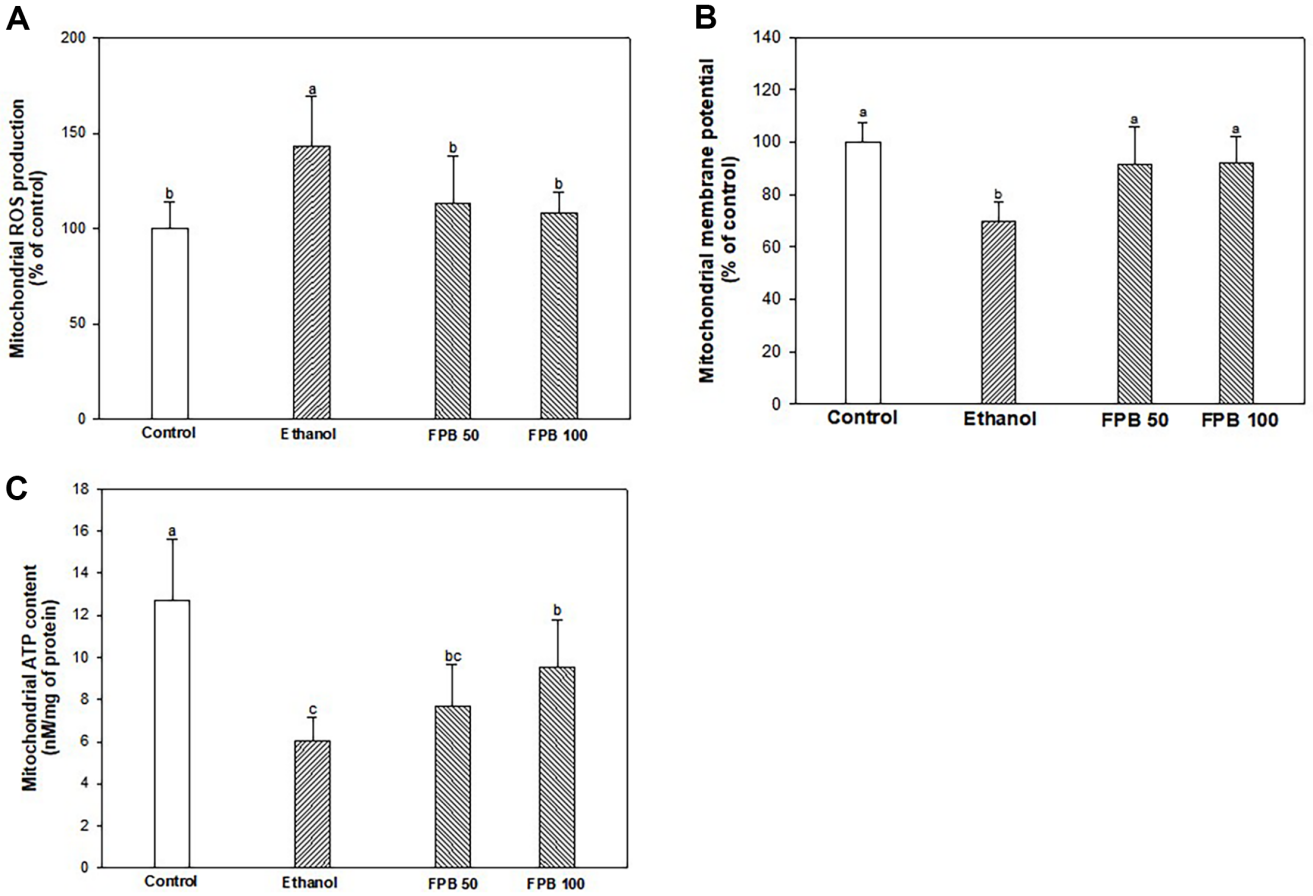
Effect of fermented *Protaetia brevitarsis* larvae (FPB) on mitochondrial function in ethanol-induced mice. Mitochondrial reactive oxygen species (ROS) production (**A**), mitochondrial membrane potential (**B**), and mitochondrial ATP content (**C**) in liver tissues. Results are presented as mean ± SD (*n* = 5). Data were statistically considered at *p* < 0.05, and different small letters represent the statistical differences.

As shown in Fig. 5A, ROS production was increased in the ethanol group (143.55%) compared with control group (100.00%). However, the ROS production of the FPB groups (FPB50, 113.44%; FPB100, 108.05%) were decreased compared with ethanol group. mitochondrial membrane potential level was reduced in the ethanol group (68.85%) compared to control group (100.00%). However, the mitochondrial membrane potential of the FPB groups (FPB50: 91.79%; FPB100: 91.94%) were increased compared with ethanol group (Fig. 5B). ATP content was reduced in the ethanol group (6.02 nM/mg of protein) compared to the control group (12.73 nM/mg of protein). However, the ATP content of the FPB100 group (9.53 nM/mg of protein) was increased compared with the ethanol group (Fig. 5C).

### Effect of FPB on Alcohol Metabolism Pathway

**Fig. 6:**
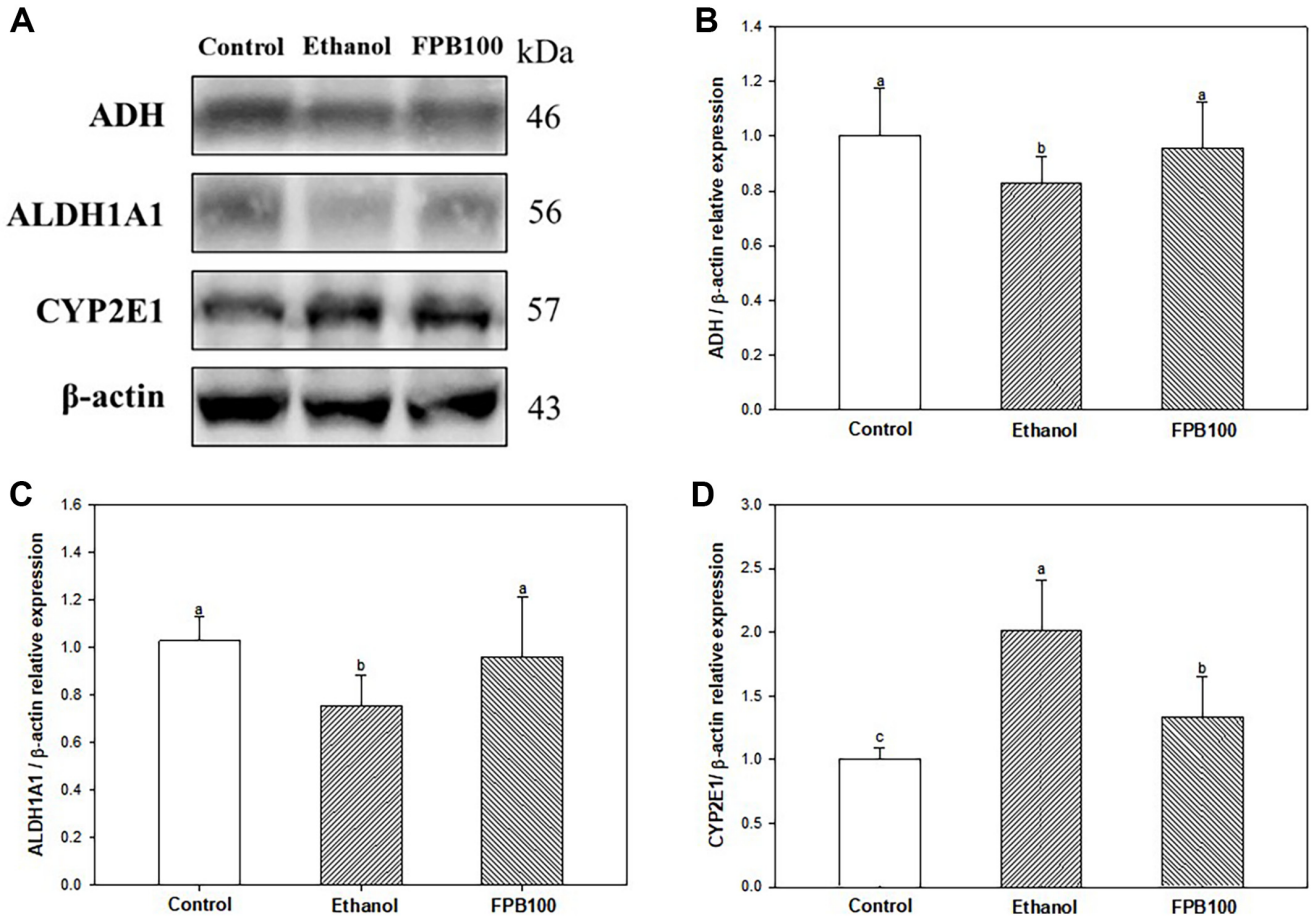
Effect of fermented *Protaetia brevitarsis* larvae (FPB) on alcohol metabolism pathway in ethanolinduced mice. Western blot band images (**A**). Relative expressions of ADH (**B**), ALDH (**C**), and CYP2E1 (**D**) on the corresponding quantitation to β-actin. Results are presented as mean ± SD (*n* = 3). Data were statistically considered at *p* < 0.05, and different small letters represent the statistical differences.

As shown in Fig. 6, the expressions of ADH (Fig. 6A and 6B) and ALDH1A1 (Fig. 6A and 6C) were decreased in the ethanol group (0.83 and 0.76, respectively) compared with the control group (1.00). However, the expressions of ADH and ALDH1A1 in the FPB100 group (0.96 and 0.96, respectively) were increased compared with the ethanol group. The expression of CYP2E1 (Fig. 6A and 6D) was increased in the ethanol group (2.02) compared with the control group (1.00). However, the expression of CYP2E1 in the FPB100 group (1.33) were reduced compared with the ethanol group.

### Effect of FPB on Apoptosis Pathway

**Fig. 7:**
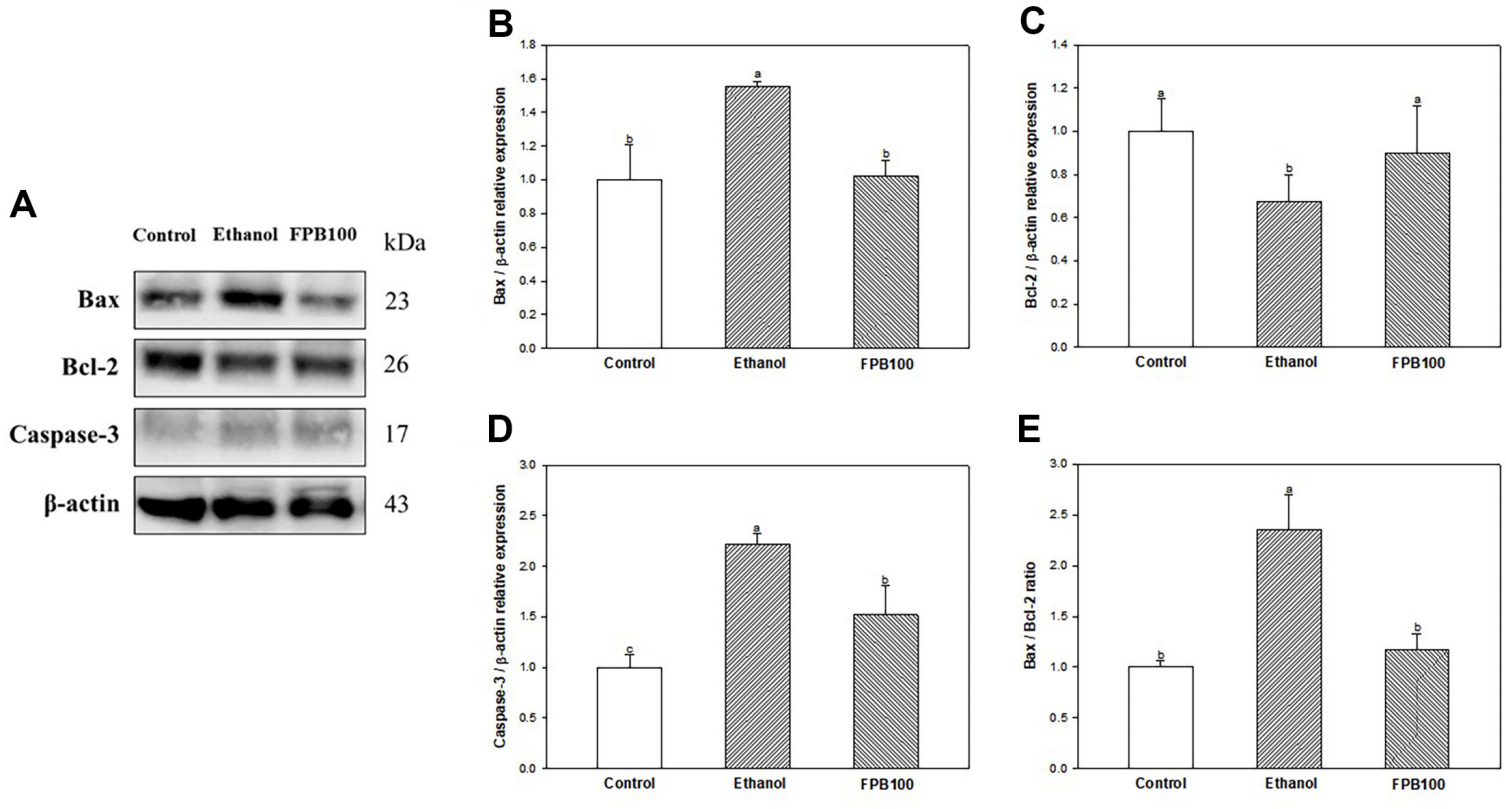
Effect of fermented *Protaetia brevitarsis* larvae (FPB) on apoptosis pathway in ethanol-induced mice. Western blot band images (**A**). Relative expressions of Bax (**B**), Bcl-2 (**C**), Bax/Bcl-2 ratio (**D**), and caspase-3 (**E**) on the corresponding quantitation to β-actin. Results are presented as mean ± SD (*n* = 3). Data were statistically considered at *p* < 0.05, and different small letters represent the statistical differences.

As shown in Fig. 7, the expression of Bax (1.55) (Fig. 7A and 7B) was increased, while the expression of Bcl-2 (0.67) (Fig. 7A and 7C) was reduced in the ethanol group compared with the control group (1.00).

In contrast, the expression of Bax (1.02) was decreased and the expression of Bcl-2 (0.90) was increased in the FPB 100 group compared with the ethanol group. Bax/Bcl-2 ratio (Fig. 7E) and expression of caspase-3 (Fig. 7A and 7D) were increased in the ethanol group (2.36 and 2.21, respectively) compared with the control group (1.00). However, the Bax/Bcl-2 ratio and expression of caspase-3 were decreased in FPB100 group (1.17 and 1.52, respectively) compared with ethanol group.

### Effect of FPB on Lipid Metabolism Pathway

**Fig. 8:**
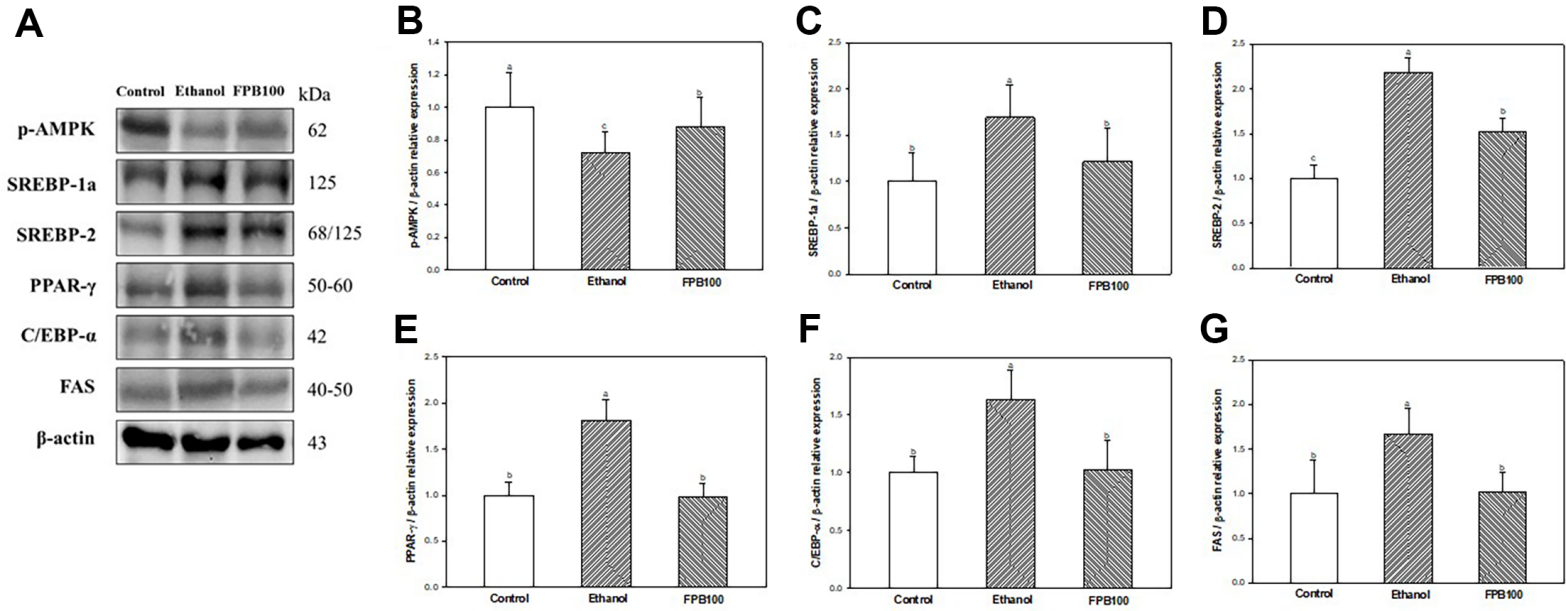
Effect of fermented *Protaetia brevitarsis* larvae (FPB) on lipid accumulation metabolism pathway in ethanol-induced mice. Western blot band images (**A**). Relative expressions of p-AMPK (**B**), SREBP-1a (**C**), SREBP-2 (**D**), PPAR-γ (**E**), C/EBP-α (**F**), and FAS (**G**) on the corresponding quantitation to β-actin. Results are presented as mean ± SD (*n* = 3). Data were statistically considered at *p* < 0.05, and different small letters represent the statistical differences.

As shown in Fig. 8, the expression of p-AMPK (Fig. 8A and 8B) was decreased in the ethanol group (0.72) compared with the control group (1.00). However, the expression of p-AMPK in the FPB100 group (0.88) was increased compared with the ethanol group. In contrast, the expressions of SREBP-1a (Fig. 8A and 8C), SREBP-2 (Fig. 8A and Fig. 8D), PPAR-γ (Fig. 8A and 8E), C/EBP-α (Fig. 8A and 8F), and FAS (Fig. 8A and 8G) were increased in the ethanol group (1.70, 2.18, 1.81, 1.63, and 0.67, respectively) compared with the control group (1.00). However, the expression of SREBP-1a, SREBP-2, PPAR-γ, C/EBP-α, and FAS in the FPB100 group (1.22, 1.52, 0.98, 1.03, and 1.01, respectively) were reduced compared with the ethanol group.

### Effect of FPB on Inflammatory Pathway

**Fig. 9:**
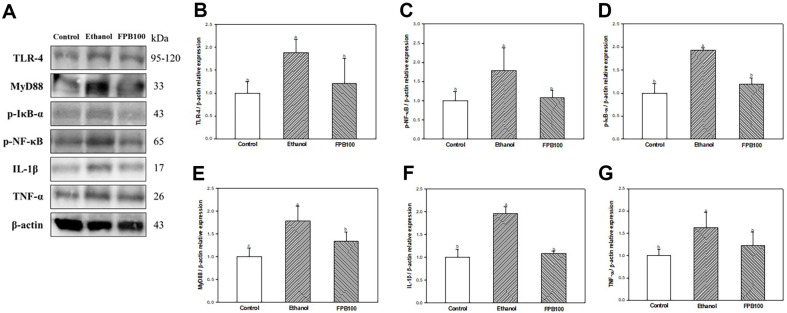
Effect of fermented *Protaetia brevitarsis* larvae (FPB) on inflammatory pathway in ethanol-induced mice. Western blot band images (**A**). Relative expressions of TLR-4 (**B**), MyD88 (**C**), p-IκB-α (**D**), p-NF-κB (**E**), IL-1β (**F**), and TNF-α (**G**) on the corresponding quantitation to β-actin. Results are presented as mean ± SD (*n* = 3). Data were statistically considered at *p* < 0.05, and different small letters represent the statistical differences.

As shown in Fig. 9, the expressions of TLR-4 (Fig. 9A and Fig. 9B), MyD88 (Fig. 9A and 9C), p-IκB-α (Fig. 9A and 9D), p-NF-κB (Fig. 9A and 9E), IL-1β (Fig. 9A and 9F), and TNF-α (Fig. 9A and 9G) were increased in the ethanol group (1.88, 1.79, 1.93, 1.79, 1.96, and 1.63, respectively) compared with the control group (1.00). However, the expression of TLR-4, MyD88, p-IκB-α, p-NF-κB, IL-1β, and TNF-α in the FPB100 group (1.20, 1.34, 1.19, 1.08, 1.08, and 1.22, respectively) were reduced compared with the ethanol group.

### Effect of FPB on Fibrotic Pathway

**Fig. 10:**
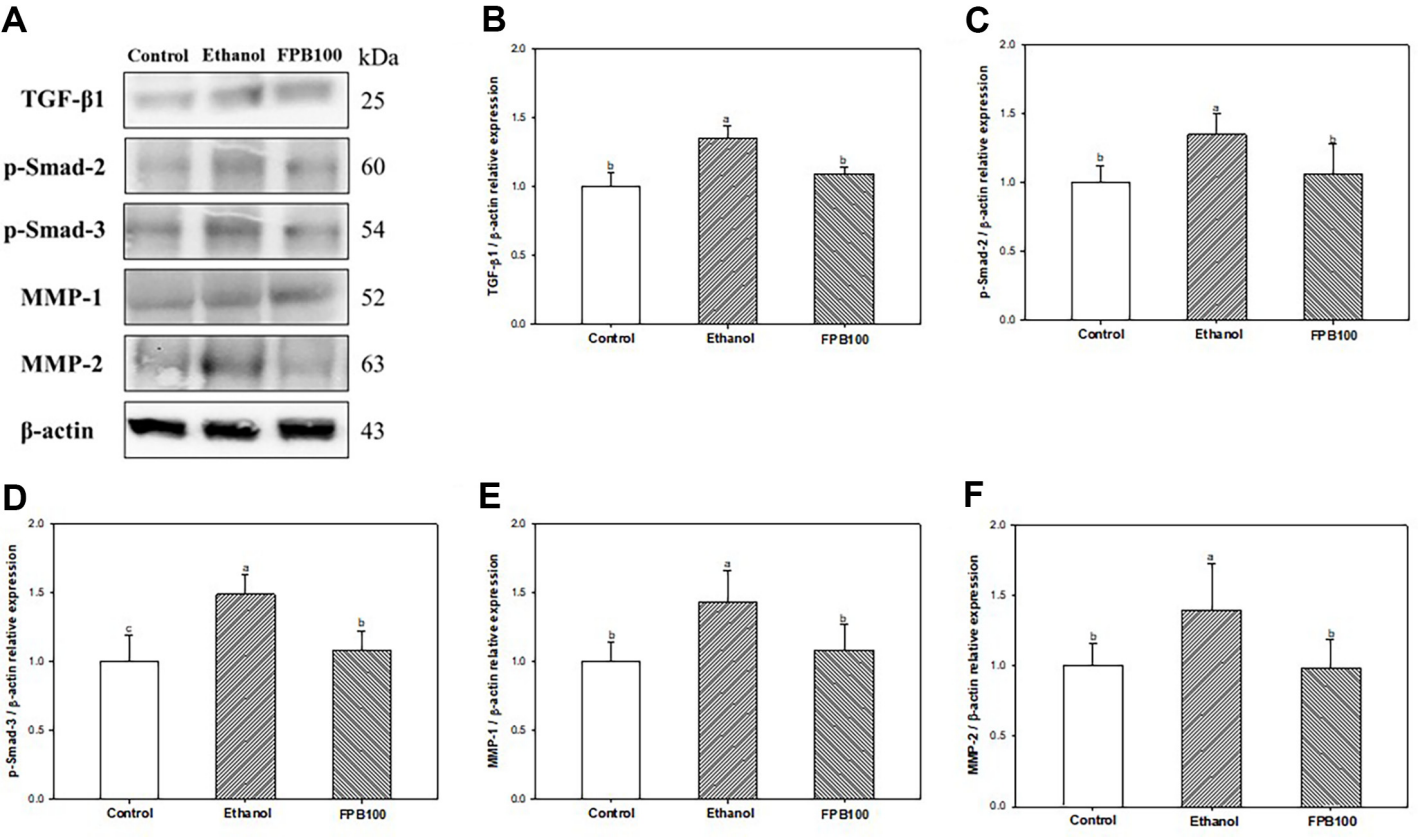
Effect of fermented *Protaetia brevitarsis* larvae (FPB) on fibrotic mediators in ethanol-induced mice. Western blot band images (**A**). Relative expressions of TGF-β1 (**B**), p-Smad-2 (**C**), p-Smad-3 (**D**), MMP-1 (**E**), and MMP-2 (**F**) on the corresponding quantitation to β-actin. Results are presented as mean ± SD (*n* = 3). Data were statistically considered at *p* < 0.05, and different small letters represent the statistical differences.

As shown in Fig. 10, the expressions of TGF-β1 (Fig. 10A and 10B), p-Smad-2 (Fig. 10A and 10C), p-Smad-3 (Fig. 10A and 10D), matrix metalloproteinase (MMP)-1 (Fig. 10A and 10E), and MMP-2 (Fig. 10A and 10F) were increased in the ethanol group (1.35, 1.35, 1.49, 1.44, and 1.39, respectively) compared with the control group (1.00). However, the expression of TGF-β1, p-Smad-2, p-Smad-3, MMP-1, and MMP-2 in the FPB100 group (1.10, 1.06, 1.08, 1.08, and 0.98, respectively) were reduced compared with the ethanol group.

## Discussion

Chronic excessive alcohol consumption can lead to ALD, including hepatic fibrosis, steatohepatitis, and liver fibrosis. Alcoholic oxidative damage can cause mitochondrial dysfunction, cell death, lipid metabolism and inflammation that promote the development of ALD. Therefore, early control of liver function before hepatitis occurs can be an important point in preventing the progression of ALD. Although various physiological activity effects of *P. brevitarsis* larvae have been reported [14-17], the mechanism of improving ALD is unknown. Therefore, this study was conducted to assess the utility of FPB as a preventive strategy for ALD by confirming the hepaprotective effect on chronic ethanol consumption mice.

Edible insects are a complete protein source known to contain essential amino acid levels similar to soybeans [28]. In addition to being a great source of protein, they contain unsaturated fatty acid, dietary fiber, essential minerals, and vitamins [29]. Studies on the nutritional content and safety evaluation of *P. brevitarsis* larvae according to the processing method have already been performed [15, 16]. However, there is little research on chemical components that have physiological activity and their functionality in *P. brevitarsis* larvae. As a result of analyzing the composition of 18 amino acids of FPB, the composition of glutamic acid, lysine, proline, arginine, and aspartic acid was the highest in this study (Table 2). In addition, the content of branched-chain amino acids (BCAAs) containing leucine, isoleucine, and valine, is generally known to be less distributed in vegetable proteins [30], but its high content was detected in FPB. In addition to being essential for cellular protein synthesis, BCAAs have several physiological regulatory functions in the body such as promoting muscle synthesis and lipolysis and helping to strengthen the immune system [31, 32]. It was confirmed that the total amino acid content of FPB was 407.32 mg/g, higher than the total amino acid content of aqueous, ethanol, and methanol extracts of *P. brevitarsis* larvae examined in a previous study [18]. Furthermore, the BCAA content of FPB was 130.29 mg/g, which was higher than that of *P. brevitarsis* larvae protein hydrolysates confirmed in a prior study [33]. These results suggest that *P. brevitarsis* larvae fermented with *B. subtilis* KCTC 1428BP after protein hydrolysis used in this study have significantly higher amino acid content than solvent extracts and protein hydrolysis or fermentation extracts single-handedly.

ADH considered the major important enzyme in alcohol metabolism, is known to have reduced activity in ALD patients [34]. When there is excessive alcohol or chronic alcohol consumption, the activity of the MEOS enzyme system increases to eliminate alcohol [35]. Therefore, a reduction of ADH activity increases the proportion of alcohol metabolism by the CYP2E1 enzyme, which can contribute to ROS accumulation in the liver [36]. Activation of MEOS makes alcoholics resistant to alcohol abuse and can convert other drugs to extremely toxic substances [37]. Acetaldehyde is known as a toxic substance based on strong reactivity and causes lipid peroxidation and DNA damage [38]. In addition, acetaldehyde impairs cell function and gene expression by covalently binding to various proteins in the liver and altering liver function and structure [39]. However, acetaldehyde can be converted into a stable and non-toxic compound by the sulfhydryl group of cysteine [40]. Therefore, it seems that cysteine contained in FPB can improve liver toxicity caused by acetaldehyde. In previous in vitro study, it was confirmed that *P. brevitarsis* larvae extract increased the activities of ADH and ALDH [41]. Moreover, in a prior study it was reported that leucine can accelerate ethanol elimination after acute ethanol administration by enhancing the activity of alcohol metabolizing enzymes such as ADH and ALDH in rats [42]. In this study, it was shown that the intake of FPB before ethanol treatment significantly reduces the serum alcohol and acetaldehyde concentration (Fig. 3). In addition, FPB enhanced ADH and ALDH expressions, and decreased CYP2E1 expression in liver tissue as well as increased in vitro ADH and ALDH activities (Figs. 1 and 6). These results suggest that FPB, which is rich in amino acids such as leucine, prevents rapid rises in serum ethanol and acetaldehyde concentrations by regulating the activities of ADH, ALDH, and CYP2E1.

Most alcohol are oxidized by ADH, but chronic alcohol consumption increases the CYP2E1 pathway and generates reactive products such as acetaldehyde and ROS [7]. ROS, which are produced concurrently with alcohol metabolism, is considered to be a major cause of proceeding ALD [3]. Excess ROS causes protein oxidation and lipid peroxidation, generating MDA and increasing the permeability of cell membrane structures [42]. As a result, GOT and GPT, which indicate liver injury, can be released into the circulation [43]. Excessive alcohol consumption can destroy the antioxidant system in the liver by reducing the level of antioxidant enzymes such as SOD and GSH [44]. In a previous study, *P. brevitarsis* larvae extract intake ameliorated liver function by decreasing CYP2E1 enzymes activity and MDA content in ethanol-induced rats [45]. Several studies have confirmed that proline has antioxidant effects by acting as a singlet oxygen scavenger with ROS scavenging activity [46, 47]. A previous study has shown that protein hydrates of *P. brevitarsis* larvae reduce H_2_O_2_-induced oxidative stress in AML12 cells (hepatocytes) by regulating the Nrf2/HO-1 signaling pathway [33]. Moreover, protein extracts of *P. brevitarsis* larvae showed reduced ROS production against H_2_O_2_-induced oxidative stress in C2C12 myoblast [48]. Also in a prior study, supplementation with BCAAs in-creased the mRNA levels of glutathione peroxidase 1 (GPX1), catalase, and SOD in the liver tissue of alcohol-induced rats [49]. Research has reported that the availability of amino acids including cysteine, arginine, glutamine, and glycine play an important role in the antioxidant defense system by contributing to GSH synthesis [50]. In this study, consumption of FPB improved the ethanol-damaged antioxidant defense system (Fig. 4). In addition, this study showed that FPB reduced the levels of liver damage markers such as GOT, GPT, TBIL, and LDH in serum that increased due to oxidative stress (Table 3). Because of this, it is considered that FPB can protect the antioxidant system by contributing to GSH synthesis and reducing intracellular oxidative stress and can improve serum biomarkers against ethanol-induced liver injury. Consequently, the current results suggest that amino acid-rich FPB can be used as a defense against ethanol-induced oxidative stress.

Mitochondria are important organelles for cellular energy production and reactive species formation, and mitochondrial hypertrophy and impaired function are hallmarks of ALD [2]. Acute and chronic ethanol administration has been reported to increase superoxide production in hepatic mitochondria, which can produce aggressive ROS, leading to alterations in mitochondrial function and structure [51]. Moreover, chronic ethanol consumption is sufficient to cause mitochondrial damage by inducing an imbalance in intracellular redox states [52]. Because ethanol metabolism reduces NAD^+^ to NADH, the cellular NAD^+^/NADH redox ratio is decreased, and accumulated NADH gives electrons to the electron transport chain in the inner membrane of the mitochondria to produce more ROS in early stages of alcohol consumption [53]. In addition, when there is a lack of capability to remove excessively generated ROS, mitochondrial DNA damage and division are caused by reducing mitochondrial membrane potential [54]. When mitochondria are damaged by ethanol, more reactive oxygen is discharged, and as a result, a vicious cycle can repeat by decreased ATP production. In summary, ethanol-mediated mitochondrial damage can destroy normal cellular activity by reducing ATP production due to mitochondrial membrane potential reduction, in tandem with excessive accumulation of ROS. A previous study has shown that arginine supplementation ameliorated H_2_O_2_-induced ovine intestinal epithelial cells damage to by enhancing TCA cycle activity and mitochondrial function [55]. Moreover, L-carnitine not only helps restore protein synthesis in the mitochondrial membrane, but also improves morphology [56]. Carnitine, which contains the basic amino acids lysine, methionine, and NH_4_^+^, is known to an important substance for transporting fatty acids into mitochondria and converting them into energy [57]. Moreover, BCAAs supplementation prevented increases in the NAD^+^/NADH ratio and mitochondrial damage in the liver tissue of alcohol-induced ALD rats [34]. Therefore, it is speculated that FPB containing lysine and methionine can induce the synthesis of L-carnitine to maintain mitochondrial function. In fact, it was confirmed in this study that mitochondrial ROS content increased along with the decrease of mitochondrial ATP and mitochondrial membrane potential in the ethanol group, and these results were effectively improved by FPB pretreatment (Fig. 5). Thus, these results suggest that FPB, a diet rich in amino acids such as arginine, lysine, and methionine, can ameliorate alcohol-induced mitochondrial dysfunction.

Alcohol-induced oxidative damage leads to a rapid drop in ATP production, which is likely to lead to apoptosis [58]. Mitochondrial dysfunction by oxidative stress is responsible for decreased mitochondrial membrane potential and the onset of mitochondria permeability transition [58]. Through this, the pro-apoptotic proteins, Bax and Bcl-2 homologous antagonist killer (Bak) can permeabilize the outer mitochondrial membrane, inducing translocation of cytosol to mitochondria [59]. Subsequently, mitochondrial membrane destruction initiates apoptosis by inducing the release of cytochrome c into the mitochondrial cytosol, activating the caspase pathway [60]. In a previous study, ethanol treatment increased apoptosis by ethanol-mediated cytochrome c/caspase-3 activation pathway in hepatocytes [61]. On one hand, in an in vitro study, it was reported that the protein extract of *P. brevitarsis* larvae showed a cytoprotective effect by decreasing the pro-apoptotic protein Bax and increasing the anti-apoptotic protein Bcl-2 in H_2_O_2_-induced C2C12 myoblast [46]. This research is consistent with the results of the protective effect of FPB against ethanol- and H_2_O_2_-induced cytotoxicity on HepG2 cells in the present study. In this regard, it was also verified that the ingestion of FPB protected the activation of the ethanol-induced apoptosis pathway (Fig. 7). These results suggest that FPB can prevent liver apoptosis by improving against mitochondrial dysfunction in the liver, and consequently contribute to the improvement of ethanolic liver injury.

Alcoholic fatty liver is an early event in ALD, and there are several mechanisms of ethanol-induced fatty liver [1]. Ethanol direct/indirect toxicity can induce mitochondrial β-oxidation and suppress of citric acid cycle, which synthesis of fatty acid is increased, thus inducing alcoholic fatty liver [52, 53]. Also, acetaldehyde is decomposed by ALDH to produce acetate, and is converted to acetyl-CoA, thereby contributing to the synthesis of cholesterol and fatty acids [2]. In summary, lipid content in the liver is efficiently regulated by maintaining the balance between intracellular fatty acid synthesis and oxidation by AMPK [62]. However, alcohol consumption can lead to a reduction of AMPK activity by decreasing mitochondrial β-oxidation and ATP production and increasing intracellular lipid accumulation [63]. Previous studies had been shown that the treatment of ethanol significantly decreased the level of p-AMPK and total AMPK in the liver [9,64]. Moreover, inhibition of AMPK by ethanol feeding can allow an increase in SREBP activity, which is involved in fatty acid and cholesterol synthesis [64]. Studies have reported that acetaldehyde increased fatty acid synthesis by increasing the expression of SREBP-1 [65,66]. In addition, it has been reported that ethanol intake increases PPAR-γ, which is an important transcription factor for TG synthesis with C/EBP-α in liver tissue [65]. Therefore, alcohol consumption leads to fatty liver, which can create an environment in the liver that is more susceptible to alcoholic liver damage. It has been reported that the protein hydrolysates of *P. brevitarsis* larvae improved liver injury by regulating mRNA expressions of AP2/FABP4, AMPKα2, and PPAR-γ and decreasing adipocyte size in high-fat diet (HFD)-induced fatty liver mice [67]. Ahn *et al*. [15] reported that the decrease in serum lipid levels and liver lipid accumulation in obese mice fed *P. brevitarsis* larvae was attributed to oleic acid. In addition, a previous study showed that silkworm can regulate the gene expression of AMPK and SREBPs in ethanol-induced rat liver tissue, which was presumed to be due to various essential amino acids and omega-3 fatty acids contained in silkworm [68]. Similarly, in the present study, FPB fed increased the expression of p-AMPK and reduced the expression of SREBPs, PPAR-γ, and C/EBP-α and serum lipid levels (Table 4 and Fig. 8). Through this, it is assumed that the inhibitory effect of lipid accumulation in the liver of FPB in this study is due to the unsaturated fatty acids. Consequently, FPB can lower the risk of alcoholic fatty liver by decreasing serum lipid levels and regulating lipogenesis-related protein through the AMPK pathway.

Ethanol consumption causes both direct damage to hepatocytes, hepatic stellate cells (HSCs), and KCs damage and indirect damage by the propagation of inflammation [69]. KCs are macrophages residing in the liver and play an important role in the innate immune response to the influx of antigens from the intestine [70]. Chronic alcohol consumption can lead to an imbalance in the gut microbiota, which can increase the amount of endotoxin flowing into the liver [71]. Endotoxins that have entered hepatic portal circulation can be recognized by immunological receptors such as Toll-like receptors present in KCs [71]. As a result, LPS, which are bacterial toxins recognized by TLR-4 in KCs, induce the formation of the MyD88 multiprotein complex, leading to NF-κB signaling activation and consequently the release of pro-inflammatory cytokines [72]. Inflammatory cytokines are one of the mechanisms contributing to the pathogenesis of ALD, and alcohol makes liver cells more susceptible to inflammatory response-mediated cell death [35]. Several in vitro studies reported that *P. brevitarsis* larvae exhibited anti-inflammatory activity in LPS-induced RAW264.7 cells [73, 74]. In addition, *P. brevitarsis* larvae reduced inflammatory cell infiltration in the adipose tissue of HFD-induced mice [67]. In a previous study, arginine improved inflammatory changes in the liver by decreasing levels of endotoxin, MDA, and the expression of p-NF-κB, and TNF-α in ethanol-induced rats [75]. In addition, it has been reported that various fatty acids contained in *P. brevitarsis* larvae, have manifold physiological activities and protect the inflammation defense system in non-alcoholic fatty liver [14, 76]. Furthermore, the hepatoprotective activity of dietary linolenic acid was assessed by regulating the mRNA expression levels of TNF-α, IL-1β, MCP-1, and iNOS in liver tissue of chronic ethanol exposure mice [77]. It has been reported that the content of oleic acid accounts for a large proportion in *P. brevitarsis* larvae, and a previous study has established that dietary oleic acid can regulate liver X receptors to prevent liver inflammation and damage caused by lipid production [78]. Similarly, this study also confirmed that FPB containing beneficial amino acids and fatty acids ameliorated alcoholic liver inflammation by inhibiting oxidative stress as well as inflammatory reaction by TLR-4/NF-κB pathway activation (Fig. 9). Therefore, these results suggest that FPB applies anti-inflammatory effects by regulating the TLR/NF-κB pathway in the liver. Furthermore, these effects of FPB can be seen as evidence that FPB intake before alcohol administration prevents the progression of liver inflammation caused by alcohol-induced oxidative stress and intestinal-derived endotoxin.

To the increase of inflammatory response in ALD, ethanol might also cause hepatic fibrosis-related indicators such as TGF-β1, Smad, and α-smooth muscle actin (α-SMA) by activating HSCs [1]. When hepatocytes are damaged by alcohol abuse, various inflammatory cytokines and ROS are produced by the interaction between HSCs, KCs, and lymphocytes [3]. Activated HSCs and fibroblasts induce the deposition of ECM to form fibrotic tissue [73]. In addition, acetaldehyde generated by alcohol metabolism activates adjacent HSCs directly to produce pro-inflammatory cytokines and TGF-β1, and accelerates fibrosis by synthesizing collagen [5, 69]. When TGF-β1 binds to its receptor, Smad2/3, the protein indicators regulating the transcription of genes encoding collagen, are phosphorylated and bind with Smad4, followed by translocation into the nucleus where these complexes activate transcription of profibrotic genes [79]. In addition, previous studies have shown that TGF-β1 promotes the deposition of connective tissue proteins in the ECM by activating enzymes that degrade substrates such as MMPs [82, 86]. An increase in MMP-2 causes membrane damage in the space of Disse, which activates HSCs and advances liver fibrosis [80]. Indeed, MMP-1 mRNA was increased in liver tissue in cases with severe liver fibrosis or cirrhosis patients [81]. Activation of MMPs restimulates TGF-β1, activating continuous hepatic fibrosis via the TGF-β1/Smad pathway [82]. In this study, chronic alcohol exposure increased the expression of TGF-β1, Smad2/ 3, MMP-1, and MMP-2, which are essential factors for fibrosis (Fig. 10). However, the expressions of hepatic fibrosis-related factors were improved in the FPB group compared with the ethanol group in this study. Similarly, BCAAs, composed of leucine, isoleucine, and valine, regulated the expression of TGF-β1, Smad-3, α-SMA, and Smad-7 in CCl4-induced liver fibrosis rats [83]. In addition, uptake of the methionine or cysteine, which sulfur-containing amino acid, reduced the deposition of collagen and the mRNA expression of α-SMA and TGF-β in liver tissue on thioacetamide-induced fibrotic rats [84]. Similar to these studies, the abundant amino acids contained in FPB might help prevent the progression of ethanol-induced hepatic fibrosis via TGF-β1/Smad pathway in ethanol-induced mice.

In summary, this study showed that the FPB improves ethanol-induced hepatotoxicity by TLR-4/AMPK pathway. Although further studies on chemical analysis besides amino acid analysis are needed to identify the functional substances of FPB, this study demonstrated that FPB can be used as a potential preventive strategy to ameliorate ALD.

## Figures and Tables

**Table 1:** List of primary and secondary antiboies information used in this study.

Antibody	Catalog	Manufacturer
ADH	sc-133207	Santa Cruz Biotech (Dallas, TX, USA)
ALDH1A1	sc-374076	Santa Cruz Biotech
CYP2E1	CSB-PA006425EA01HU	Cusabio (Wuhan, China)
Bcl-2	sc-509	Santa Cruz Biotech
Bcl-2-associated X protein (Bax)	sc-7480	Santa Cruz Biotech
Caspase-3	CSB-PA5689A0Rb	Cusabio
AMPK	#2535	Cell Signaling Tech (Danvers, MA, USA)
Sterol regulatory element-binding protein (SREBP)-1a	sc-13551	Santa Cruz Biotech
SREBP-2	sc-13552	Santa Cruz Biotech
Peroxisome proliferator-activated receptor (PPAR)-γ	NBP2-22106	Novus Biologicals
CCAAT/enhancer binding protein (C/EBP)-α	NB600-1438	Novus Biologicals
Fatty acid synthase (FAS)	CSB-PA0635A0Rb	Cusabio
TLR-4	sc-52962	Santa Cruz Biotech
Myeloid differentiation primary response (MyD)88	sc-74532	Santa Cruz Biotech
p-Nuclear factor (NF)-κB	sc-136538	Santa Cruz Biotech
p-NF-κB inhibitor (IκB)-α	sc-8404	Santa Cruz Biotech
TNF-α	sc-33639	Santa Cruz Biotech
IL-1β	sc-515598	Santa Cruz Biotech
Transforming growth factor (TGF)-β1	sc-130348	Santa Cruz Biotech
p-Small mothers against decapentaplegic (Smad)-2	#3108	Cell Signaling Tech
p-Smad-3	sc-517575	Santa Cruz Biotech
matrix metalloproteinase (MMP)-1	sc-21731	Santa Cruz Biotech
MMP-2	sc-13595	Santa Cruz Biotech
β-actin	66009-1-Ig	Proteintech (Rosemont, IL, USA)
Goat-anti-rabbit IgG	#7074	Cell Signaling Tech
Goat-anti-mouse IgG	#1721011	Bio-Rad

**Table 2:** Total amino acid profile and amino acid score (AAS) of protein hydrolysate from *Protaetia brevitarsis* larvae fermented by *Bacillus subtilis* (FPB).

	FPB (mg/g)	AAS*
Histidine	12.02 ± 1.35	165.00 ± 1.73
Isoleucine	14.40 ± 1.60	99.67 ± 1.53
Leucine	22.52 ± 2.46	80.00 ± 2.00
Lysine	40.73 ± 6.91	188.33 ± 5.51
Threonine	14.24 ±1.44	129.67 ± 3.06
Tryptophan	5.24 ± 0.98	186.33± 8.08
Valine	21.14 ± 2.23	112.00 ± 1.00
Methionine	5.09 ± 0.71	-
Arginine	35.18 ± 6.93	-
Phenylalanine	15.65 ± 1.63	-
Tyrosine	27.12 ± 6.84	-
Glycine	24.42 ± 2.26	-
Serine	23.42 ± 2.92	-
Alanine	18.64 ± 2.01	-
Glutamic acid	52.89 ± 4.78	-
Aspartic acid	29.72 ± 2.92	-
Proline	47.42 ± 1.84	-
Cysteine	5.88 ± 1.44	-
Total amino acid	407.32 ± 62.32	

**Table 3:** Effect of fermented *Protaetia brevitarsis* larvae (FPB) on glutamic oxaloacetic transaminase (GOT), glutamine pyruvic transaminase (GPT), gamma-glutamyl transferase (GGT), total bilirubin (TBIL), and lactate dehydrogenase (LDH) in serum of ethanol-induced mice.

Groups	GOT (U/L)	GPT (U/L)	GGT (U/L)	TBIL (mg/dL)	LDH (U/L)
Control	43.40 ± 2.51^b^	31.20 ± 3.27^b^	1.00 ± 0.00^a^	0.23 ± 0.05^b^	127.75 ± 18.61^c^
Ethanol	55.60 ± 11.52^a^	40.60 ± 5.55^a^	1.00 ± 0.00^a^	0.60 ± 0.20^a^	239.67 ± 24.85^a^
FPB 50	42.40 ± 2.19^b^	27.40 ± 3.36^b^	1.00 ± 0.00^a^	0.25 ± 0.05^b^	165.50 ± 29.85^b^
FPB 100	42.40 ± 1.82^b^	28.40 ± 1.67^b^	1.00 ± 0.00^a^	0.23 ± 0.05^b^	133.25 ± 16.28^c^

The results are presented as mean ± SD (*n* = 5). Data were statistically considered at *p* < 0.05, and different superscript small letters represent the statistical differences.

**Table 4:** Effect of fermented *Protaetia brevitarsis* larvae (FPB) on triglyceride (TG), total cholesterol (TCHO), high density lipoprotein cholesterol (HDLC), low density lipoprotein cholesterol (LDLC), and HDLC and TCHO ratio (HTR) in serum of ethanol-induced mice.

Groups	TG (mg/dL)	TCHO (mg/dL)	HDLC (mg/dL)	LDLC (mg/dL)	HTR (%)
Control	147.20 ± 22.61^ab^	109.80 ± 9.85^a^	86.40 ± 8.26^ab^	52.84 ± 4.16^b^	78.41 ± 0.69^d^
Ethanol	161.40 ± 16.53^a^	116.80 ± 10.80^a^	88.40 ± 6.11^ab^	60.68 ± 3.67^a^	75.83 ± 2.37^e^
FPB 50	113.00 ± 24.40^c^	90.80 ± 13.77^c^	74.40 ± 12.93^c^	39.00 ± 4.99^c^	81.76 ± 2.62^c^
FPB 100	126.00 ± 15.18^bc^	97.80 ± 15.93^b^	82.80 ± 11.78^b^	40.20 ± 6.80^c^	84.87 ± 2.45^a^

The results are presented as mean ± SD (*n* = 5). Data were statistically considered at *p* < 0.05, and different superscript small letters represent the statistical differences.
